# Age-Associated B Cells (ABCs) in the Prognosis, Diagnosis and Therapy of Systemic Lupus Erythematosus (SLE)

**DOI:** 10.31138/mjr.31.3.311

**Published:** 2020-09-30

**Authors:** Athanasios Sachinidis, Konstantinos Xanthopoulos, Alexandros Garyfallos

**Affiliations:** 1 Department of Pharmacognosy-Pharmacology, School of Pharmacy, Aristotle University of Thessaloniki, Thessaloniki, Greece; 2 4^th^ Department of Internal Medicine, Hippokration General Hospital, School of Medicine, Aristotle University of Thessaloniki, Thessaloniki, Greece

**Keywords:** ABCs, age-associated B cells, DN2, SLE, T-bet, autoimmunity

## Abstract

The term “age-associated B cells” (ABCs) refers to a heterogeneous B cell subset (CD19^+^,CD21^−^, CD11c^+^,T-bet^+^) which is expanded in the elderly, but also accumulates prematurely in patients with autoimmune disorders and/or infectious diseases. In healthy individuals, ABCs represent a low prevalence population that positively impacts immunosenescence. In autoimmunity and infections though, ABCs expand dramatically and produce high titers of antibodies, thus playing a role in the regulation of humoral responses. Despite the fact that these observations were made on both mice and humans, the functional features of ABCs and their exact role in human health and disease are still elusive. This review focuses on ABC and ABC-like sub-populations found in Systemic Lupus Erythematosus (SLE) patients (such as the double negative 2;DN2 population: CD19^+^,IgD^−^,CD27^−^, CXCR5^−^,T-bet^+^) and broaches the subject of their potential use as prognostic and/or diagnostic markers. The identification of novel biomarkers, via correlating the cell populations with the clinical profile of the patients, should enable better patient stratification and monitoring. Moreover, the necessity and importance of elucidating the role of transcription factor T-bet (TBX21) in the pathogenesis of human autoimmunity are addressed. T-bet, whose expression is upregulated in both mouse and human ABCs, is considered to play a major role in various aspects of autoimmunity, such as the production of autoreactive IgG, the enhanced antigen presentation to T cells and also the formation of spontaneous germinal centres (GC). Shedding light to its role in human disease, in conjunction with the characterisation of genes and pathways associated with the transcription factor itself, may lead to the discovery of novel druggable targets.

## INTRODUCTION

Systemic lupus erythematosus (SLE) is a severe autoimmune disease whose aetiology remains undefined, although B cell dysfunction seems to play a central role in its pathogenesis.^1^ More specifically, the activation of autoreactive B cells leads to the generation of pathogenic antibody-secreting cells (ASC) resulting in production of autoantibodies. The activation of the B cells involves both follicular (GC reactions) and extrafollicular pathways.^2^

A novel B cell subset, termed age-associated B cells (ABCs), was first described in 2011 by Hao et al. and Rutsov et al., as a B cell population which is expanded in the elderly, but also accumulates prematurely in patients with autoimmune disorders, such as rheumatoid arthritis (RA) and SLE.^3,4^ Since then, the ABCs have been the focus of great interest to many other research groups. In this review, we focus on the potential use of these cells as prognostic and/or diagnostic markers in SLE, as well as the possibility of targeting them for therapeutic interventions.

## AGE-ASSOCIATED B CELLS (ABCs): MAIN FEATURES AND FUNCTIONS

As already mentioned, ABCs were first described by Hao et al. and Rubtsov et al. as a B cell population, defined as B220^+^CD19^+^ splenocytes, that lack expression of CD21 and CD23 while expressing CD11c at high levels. This population expands in the healthy elderly individuals, but also displays a premature accumulation in mostly female patients with RA or SLE. ^3,4^ Of note, TLR7*,* an important gene for ABC biology, is X-linked, possibly leading to a pronounced accumulation of ABCs in females.^4^ It was later revealed that ABCs or (at least) ABC-like populations are present in other autoimmune diseases as well, including Sjögren’s syndrome,^5^ Crohn’s disease,^6^ and multiple sclerosis (MS).^7^ Moreover, ABCs and ABC-like populations are also present in various infectious diseases, such as malaria and AIDS.^8,9^ Although these cells are expected to fulfil different functions in the context of different conditions (aging, autoimmunity and infections), their exact role in each of these conditions still requires thorough investigation.

### ABCs in Healthy Subjects

In healthy individuals, ABCs represent a low -although steadily expanding-prevalence population, which is generated in response to antigenic stimulation and is functionally exhausted, thus contributing to features of immune senescence.^3^ More specifically, in age-matched healthy subjects, ABCs produce pro-inflammatory cytokines (such as TNF-α) and inhibit B lymphopoiesis via targeting for apoptosis the pro-B cells with high surrogate light chain (SLC) levels.^3,10,11^ In addition to these effects, ABCs favour polarisation to a Th17 inflammaging profile.^3^ However, despite the fact that all these findings identify ABCs as a B cell population that occupies an increasing proportion of the primary B-cell niche with age, their exact role in immune senescence still requires investigation.

### ABCs in Autoimmunity and Infections

In the context of chronic immune stimulation, such as in autoimmunity or viral infections, ABCs expand rapidly and produce antibodies (auto-antibodies or anti-viral IgG, depending on the case). ^4–9^ According to observations made in murine models of autoimmunity, in addition to the production of autoreactive IgG, ABCs are also implicated in the enhanced antigen presentation to T cells and the formation of spontaneous germinal centres.^4,12–14^ T-bet, a transcription factor which is highly expressed in ABCs, is considered to be a master regulator of all these processes,^12^ although recently published data indicate that functional murine ABCs can be generated, both *in vivo* and *in vitro*, in the absence of T-bet expression in B cells.^15^

ABC-like cells, defined as CD11c^+^CD21^−^ or IgD^−^CD27^−^, also appear in humans with various autoimmune disorders.^4–7^ Similar to their murine counterparts, these human ABCs express CD5 and CD86 and lack expression of CD23. However, unlike the murine ABCs, human ABCs are isotype switched.^4^ While all these findings strongly suggest a role for ABCs in humoral autoimmunity, further investigation is required to fully understand the underlying causal associations.

In infectious diseases, ABCs of similar phenotypes to those described in autoimmunity, seem to contribute to ineffective immune responses and the inability to successfully clear pathogens.^16,17^ In this setting, the role of T-bet remains unclear: despite the fact that B cell intrinsic T-bet expression is required to control chronic viral infections, its deficiency conferred survival advantage to post-influenza bacterial superinfections.^18,19^

#### The ABC immunophenotype – Heterogeneity among the ABCs

Hao et al. and Rubtsov et al. used different markers for the characterisation of ABCs, although the cells shared some key characteristics and their immunophenotyping profiles strongly overlapped.^3,4^ A decade later, consensus has not yet been reached as to which markers should be used to identify ABCs. Several groups have described ABCs and associated populations, but in every case the markers used were different. The heterogeneity of the markers used for the characterisation of ABCs in different publications was recently summarised in a review by Phalke and Marrack.^20^

Despite the differences, some pan-ABC markers exist and are used for the identification of the subset (**Table 1**). However, as it was correctly pointed out by Sanz et al., the use only of these “core” markers for the characterisation of ABCs is somewhat misleading, as such a strict population consists of multiple B cell populations (naïve B cells, memory B cells and double negative B cells).^21^

**Table 1.** Expression markers used for the characterisation of ABC phenotype.

**Age-Associated B cells**			
**ABC “core” markers**	**ABC additional markers**		
	**NAV**	**BMem**	**DNs**
CD19+, CD21^low^ or CD21−, CD11c^hi^, T-bet+	IgD+, CD27−	IgD^low^ or IgD−, CD27+	IgD−, CD27−

ABC: Age-Associated B cell; NAV: Naïve B cells; BMem: Memory B cells; DNs: Double Negative B cells.

#### Double Negative (DN) B cells: a distinct population or an ABC sub-population?

Double Negative (DN) B cells, also known as atypical memory B cells, are B cells that do not express immunoglobulin D and the memory B cell marker CD27.^22^ Similar to ABCs, in case we accept these two are distinct populations, DNs display an expansion in the elderly,^22^ but also represent a notable component of the B cell compartment in patients with autoimmune disorders and/or chronic infectious diseases.^9,23^

DNs have been further divided into two subgroups, based on the expression of follicular homing marker CXCR5.^24^ The CXCR5+ subgroup (DN1) is expanded in healthy elderly individuals and lacks T-bet expression, while the CXCR5- subgroup (DN2) expresses T-bet and is more marked in autoimmune diseases.^22,24,25^ The immunophenotyping profile markers for the characterisation of DN subgroups are listed in **Table 2**.

**Table 2.** Classification of Double Negative B cell subsets.

**Subset**	**Phenotype**	
**DNs**	CD19+, IgD−, CD27−	
	**Main marker**	**Additional markers**
**DN1**	CXCR5+	CD21+, T-bet−
**DN2**	CXCR5−	CD21−,T-bet+, CD11c+

DNs: Double Negative B cells; DN1 and DN2: subgroup 1 and subgroup 2 of the DNs, respectively.

#### Origin of the ABCs

Hao et al. noticed that ABCs get ablated by sublethal irradiation (5 Gy) and, in contrast to marginal zone (MZ) B cells and follicular (FO) B cells, their population does not recover rapidly. ^3^ This observation suggested that ABCs do not come from *de novo* production of a unique preimmune B cell that originated from a progenitor in the bone marrow, but are instead a slowly accumulating population, possibly derived from the peripheral preimmune B cell compartments. Moreover, adoptive B cell transfer studies performed by the very same group, showed that ABCs can be generated by FO B cells within a month after the transfer.^3^

Notably, a recent study by Russell Knode et al. revealed that ABCs express a diverse Ig repertoire of V_H_ and V_K_ genes, characterised by somatic hypermutations, and arise following antigen-driven activation.^26^ These results imply but do not demonstrate, a GC origin for the ABCs. Despite the fact that a GC origin is a possible (and maybe the most plausible) scenario, other routes leading to the generation of ABCs cannot be excluded (for example, establishment through homeostatic proliferation).^27^ Besides, as mentioned above, the B cell activation involves, apart from the GC reactions, extrafollicular and other GC-independent pathways.^2^ As far as extrafollicular activation pathways are concerned, the DN2 subgroup, a unique ABC-like population which is marked in African-American patients with severe SLE, is developmentally related to activated naïve (aNAV) B cells and displays extra-follicular characteristics, such as the lack of CXCR5 and CD62L.^24^ Thus, it is more likely that DN2 are not of GC origin and are not related to GC reactions.

#### ABC transcriptional signature, activation signals, and differentiation requirements

##### Data derived from animal models

A transcriptomic analysis of murine ABCs revealed that these cells are a unique population, discrete from MZ B cells, FO B cells, B1 B cells etc, that serve as plasmablasts (ASC precursors) and highly express integrin αx chain (CD11c) and transcription factor T-bet.^4^ The elevated expression of T-bet, which is considered to be (at least, according to the majority of the literature) a key molecule for the ABC functions, is the result of the synergistic triggering of their BCR receptor, a Toll Like Receptor (TLR7 or TLR9) and a third receptor (either IFNγR or IL-21 R).^12,28^

BCR, despite having no effect when applied alone, remains a receptor of importance for the ABCs, as it synergises with TLRs or CD40.^12^ The TLRs, considered to act as nucleic acid sensors (innate molecular pattern recognition system), mediate ABC activation and promote humoral autoimmunity (via TLR7), while on the other hand they can also sustain B cell tolerance, via a programmed cell death pathway (via TLR9).^29^ Of course, always in the context of TLR engagement, the signals required for the adoption of the T-bet+ ABC phenotype originate from IFNγ and IL-21 cytokines.^28^

It is also important to mention that ABCs, unlike the MZ and FO B cells, do not rely on B Lymphocyte Stimulator “BLyS” (also known as BAFF) for survival, but nonetheless express BR3 and TACI, two of the three BLyS receptors. ^3^ Thus, ABCs resemble the memory B cells in their survival requirements, as most of memory B cells are BLyS-independent. ^30^

##### Data derived from humans

Wang et al. and Jenks et al. focused on ABC-like populations, CD11c^hi^T-bet^+^ B cells and the DN2 subset respectively, that were found expanded in patients with SLE.^24,31^ Data derived from the work of these two groups, in general, shares many similarities with the data derived from mice (**Table 3**). The characteristics of both populations, in more detail, shall be described in a next section of this review (ABCs in the pathophysiology of human autoimmunity: emphasis on SLE).

**Table 3.** A comparative analysis of the ABCs found in murine and human SLE.

	**ABCs in SLE mice models**	**ABCs in SLE patients**
**Source**	Spleen ^4^	PBMCs ^4,31^
**Ig isotype**	IgM+ and/or IgD+ ^4^	IgG+ ^4^
**Expansion**	Always (∼5–10 fold change) ^4^	In some subjects only^4,24,31^
**Age-association**	Yes (% changes from month to month)^4^	No ^31^
**Response to anti-BCR/CD40**	Hypo-responsive ^12^	Unresponsive ^24^
**Response to TLRs**	Respond well to TLR7 and TLR9 ^4, 29^	Hyper-responsive to TLR7^24^
**Ab production**	High titres of Auto Abs ^4^	Correlation with various SLE Auto Abs^24, 31^
**Ag-presentation**	APCs ^13^	N/A
**Formation of GC**	Facilitation of spontaneous GC formation ^14, 34^	N/A
**BCR specificity**	Enriched specificity in auto-reactivity ^4^	Enriched in auto-reactivity^31^

Ab: Antibody; Ag: Antigen; APC: Antigen Presenting Cell; GC: germinal centre.

##### Regulation of ABCs in systemic autoimmunity

Manni et al. unravelled a molecular pathway, by which SWEF proteins - a two-member family of Rho GTP-ase regulatory proteins –regulate the ABCs in systemic auto-immunity.^32^ Using mice deficient in both SWEF proteins (SWAP-70 and DEF-6), the scientists observed that the mice developed a lupus-like disease, characterised by the increased formation of ABCs. The enhanced ABC formation was controlled by IL-21 and IRF5: the lack of SWEF, seems to lead to IRF5 dysregulation in response to stimulation with IL-21.^32^ Of note, DEF-6 serves as a genetic risk variant for human SLE,^33^ further supporting the notion that SWEF are also relevant to human SLE.

**Figure 1.**
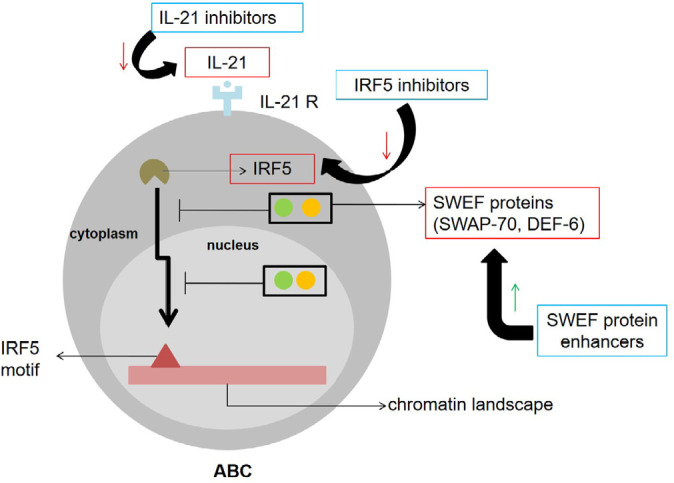
Potential therapeutic targets for the ABC-based treatment of SLE. SWEF proteins prevent the IL-21-induced transcription factor IRF5 from binding to DNA regulatory regions and recruiting transcription factor T-bet (which in its turn promotes the expansion of ABCs).^32^ Suppression of IL-21 signalling (eg, via an IL-21 R antagonist), or inhibition of IRF5 binding to IRF5 motifs (directly, via targeting of IRF5 with inhibitors or indirectly via enhancing the expression of SWEF proteins), may delay or even block ABC expansion and eventually alleviate the symptoms in SLE. *red-lined boxes: druggable targets; blue-lined boxes: potential novel therapeutics for ABC-based SLE treatment.

The elucidation of mechanisms regulating ABC, may lead to the discovery of novel therapeutic targets. Conditional targeting of the transcription factor T-bet, which is necessary and sufficient for ABC formation,^4,16^ in B cells, improves the overall health status of SLE mice, including improved kidney function, better survival rates, reduction of autoantibodies and reduced titres of serum IgG2a.^34^ Thus, in a similar way, we believe that the prevention of ABC formation (for example, via targeting and/or enhancing molecules involved in the SWEF-IRF5-IL-21 pathway) may be beneficial and have a therapeutic effect in patients with SLE (**Figure 1**).

## ABCs IN THE PATHOPHYSIOLOGY OF HUMAN AUTOIMMUNITY: EMPHASIS ON SLE

In an attempt to exemplify a role of T-bet+ B cells in human autoimmunity, Wang et al. described an unusual B cell subset (termed CD11c^hi^T-bet+ B cells) in patients with SLE, that displays many of the ABC key characteristics.^31^ In more detail, these cells express CD11c surface marker, and the transcription factor T-bet, expand in patients with SLE and also differentiate into autoreactive ASC in an IL-21 dependent manner.^31^ Moreover, similar to ABCs, these cells are BAFFR^+^ and TACI+.^3,31^

At the clinical level, interesting observations have also been made. The CD11c^hi^T-bet+ B cells seem to correlate with the percentages of various anti-nuclear autoantibodies associated with SLE (dsDNA, nucleosome, RNP, Smith etc),^31^ though there is a high probability for other B cell populations to also show such significant correlations. Further, these cells are expanded in patients with SLE compared to healthy individuals, and the expansion correlates with the severity of clinical manifestations.^31^ More specifically, the disease activity index (SLEDAI) of a patient, correlates with the frequency of the cells found. Notably, in the case of similar SLEDAI scores among patients, the cell frequency can be associated with distinct disease manifestations, such as active nephritis, indicating the specificity of this clinical association. ^31^

A transcriptomic analysis performed by Wang et al. revealed that CD11c^hi^T-bet+ B cells sorted from both patients with SLE and RA, share similar traits, as the expression patterns of cytokines and/or cytokine receptors, transcription factors, migration molecules and signalling factors coincide.^31^ However, in comparison to healthy control individuals, the majority of the genes does not share a similar pattern, indicating that the functions of these cells change dramatically in autoimmunity.^31^

Surprisingly, despite all the similarities with typical ABCs, the CD11c^hi^T-bet+ B cell subset does not increase with age, indicating that it may actually not be an age-associated population.^31^ In fact, in cases of SLE and independent of the patient’s sex, these cells tend to decrease with age (though the decrease is more marked in males) and in cases of healthy subjects, no age-correlation is evident.^31^ Considering the fact that increasing age-associated B cells have been characterised phenotypically in females with RA as a class-switched memory B cell subset,^4^ we believe that the CD11c^hi^T-bet B cell subset may in fact be a different sub-population. It is important to note that consistent with the observations by Wang et al., B cells of similar phenotype - and not age-associated - have also been identified in healthy individuals and MS patients.^7,35^

Another interesting work, focusing on the DN2 subset in SLE, was carried out by Jenks et al.^24^ Similarly to previous reports, DN2 cells in this work lack the expression of IgD and CD27 markers, but also the expression of homing marker CXCR5 (which indicates the extrafollicular origin of this subset and actually differentiates it from DN1).^24^ Its activation is mediated by hyper-responsiveness to TLR7 and leads, after the cooperation of IL-21 and also IFNγ, to the generation of autoreactive ASC.^24^ DN2s are characterised by a unique expression pattern of cytokines and cytokine receptors, transcription factors, signalling factors and others, indicating that these cells are distinct from other B cell populations.^24^

Expansion of DN2 cells is particularly evident in African American patients with severe SLE. The subset often becomes the predominant population of B cells in active SLE patients, characterised by active nephritis and high lupus autoantibody titres.^24^ Similar to CD11c^hi^T-bet+ B cells, the DN2s do not expand continuously with age, as the highest frequencies of these cells are reported in young patients.^24^ Despite the fact that the DN2 subset was presumed to be SLE-specific, a recent study by Richardson et al. implicated the cells in the pathogenesis of common variable immunodeficiency (CVID).^36^

Double negative (DN) B cells had been implicated in the pathogenesis of SLE, long before DN1 and DN2 subsets were described.^23^ The deeper characterisation of these cells, via immunophenotyping, has the potential to identify novel SLE phenotypes that associate with disease-activity. Hritzo Ahye MK and Golding A identified such a phenotype, based on the localisation of FOXO1 transcription factor.^37^ FOXO1, which is involved in B cell development,^38^ translocates from the nucleus to the cytoplasm (presumably in an AKT-dependent manner) in response to BCR ligation, and thus gets inactivated.^39^ In SLE, a cytoplasmic FOXO1 double negative B cell population – termed CytoFOX DN B cells – expands in patients, with the expansion being more pronounced in African-American females.^37^ In fact, the DN B cells of the patients are enriched in CytoFOX DNs. These cells seem to correlate directly with SLE activity, implying that the cytoplasmic localisation of FOXO1 may represent a novel biomarker of the disease progression.^37^

Whether CytoFOX DNs constitute a novel DN population or belong to the DN2 subset (the DN1 is not associated with autoimmunity after all),^24^ is an issue that requires further investigation. We believe that there is a strong possibility for these cells to be a sub-population of the DN2 subset, as the highest frequencies of them have been observed once again in African American females with high SLEDAI score.^37^ However, we cannot also rule out the possibility that a “third” DN subset also exists.

## IS “AGE-ASSOCIATED B CELLS” A PROPER DESIGNATION AFTER ALL?

In the literature, the term “age-associated B cells” is used by the authors (directly, but also circumlocutorily sometimes) to define various B cell populations, that share some key characteristics. Despite the similarities, these populations also display important differences. The CD11c^hi^T-bet+ B cells and the DN2 B cells described above,^24,31^ are only two examples of many populations characterised as ABCs or ABC-like. Considering the fact that these two specific populations do not expand continuously with age,^24,31^ we find the use of “age-associated” term misguiding. Moreover, as already pointed out by S. Phalke and P. Marrack,^20^ even in the cases of truly age-associated populations, the cells also associate with autoimmunity and infectious diseases. For this reason, the ABC acronym sometimes serves as an abbreviation for Autoimmune-Associated B cells,^20^ but even so, we believe that the term remains misguiding, as there is no reference to their role in infectious diseases.

To avoid these semantic problems, it may be a good practice to describe the populations of interest by using one or two core expression markers. For instance, the descriptor “CD11c^hi^T-bet+ B cells”,^31^ even if it refers to multiple B cell populations, is accurate enough, as it focuses on B cells that highly express CD11c marker and the transcription factor T-bet.

## ABC-BASED PROGNOSIS, DIAGNOSIS AND THERAPY OF SLE: FUTURE DIRECTIONS

It is well known that ethnicity - among other factors - is linked to the severity of SLE manifestations.^40^ By and large, the non-Caucasian populations (African, Asian and Hispanics) are more prone to suffer from severe SLE with high disease activity.^40^ A better understanding of the differences among the ethnic groups may influence the disease management.

Some ABC-like populations, such as the DN2 subset or the CytoFOX DN B cells, expand preferentially in African American patients with SLE.^24,37^ On the other hand, different ABC-like populations, such as the CD11c^hi^T-bet+ B cells, do not seem to be affected by the ethnicity of the patient.^31^ Nevertheless, all these cells correlate with high burden of disease.^24,31,37^ We thus believe that by correlating these cell populations with the clinical profiles of the patients, we may be able to identify novel prognostic and/or diagnostic markers of SLE. Notably, the cytoplasmic localisation of FOXO1 in CytoFOX DN B cells has already been associated with an SLE phenotype and its activity,^37^ enhancing even more the probability of exploiting these cells as targets of therapeutic approaches. Given that ethnicity influences the severity, symptomatology, and general pathophysiology of the disease, we believe that consideration of ethnic origins of patient is important when correlating clinical state with specific B cell sub-populations. For instance, it would be very interesting for future studies to assess whether a Mediterranean SLE population differs from an African American population, in terms of ABC immunophenotype.

In order to properly validate the prognostic and/or diagnostic biomarkers of SLE, the effects of medication, used in clinical practice, on the percentage and even the functions of ABCs (or ABC-like populations) responding to specific patient ABC profiles should also be considered, in addition to the correlation between the cell populations and the clinical state of the individuals. The ultimate goal would be to correlate ABC profiles with drug response. Such an approach will enable better patient stratification and monitoring and eventually introduce personalised medicine in SLE.

Thus far, interesting observations have been derived from a follow-up study, involving SLE patients from Sweden initiating belimumab (Benlysta, GlaxoSmith Kline), a monoclonal antibody targeting the B cell cytokine BLyS, approved for the treatment of the disease.^41^ According to the study, belimumab rapidly decreased ABC-like cells leading to significant reductions even at the first follow-up visit. DNs were also reduced, albeit at a slower rate, and the effects were evident at subsequent visits. These observations correlated with early, but not late, clinical improvements. Moreover, the high or low baseline B cell counts were predictive of success or failure respectively to attain low SLE activity.^41^ Huang W et al. made also some similar observations on a small group of SLE patients, of multi-ethnic background, and further suggested that belimumab promotes the negative selection of activated autoreactive B cells and thus mostly affects that specific pool of cells.^42^

In addition to the prognostic and/or diagnostic potential of ABCs and their relative populations, we believe that all these cells may be exploited for therapeutic purposes too. In fact, given that these cells are autoreactive and drive pathogenicity,^4,24,31^ it is reasonable to believe that their targeting and their inactivation shall be beneficial. Of course, in order to successfully do so, it is necessary to fully understand the underlying mechanisms by which ABCs and ABC-like cells develop and function. To this end, we focus on transcription factor T-bet, which seems to serve as a master regulator of these cells.^12^

T-bet is a transcription factor which mainly serves as a Th1 lineage commitment regulator,^43^ but is also expressed in other cell types such as NK cells, dendritic cells and B cells, and orchestrates their function in the immune system.^44–46^ In the B cells, T-bet is responsible for isotype switching to IgG2a (in mice) and the appearance of IgG2a-expressing memory B cells.^47, 48^ In the case of ABCs, more specifically, T-bet expression seems to play a critical role in both autoimmunity and infections,^4,12,16^ though other studies indicate that the role of T-bet is dispensable.^15,19^ These contradictory findings render the elucidation of T-bet’s role, and the identification of genes and pathways regulated by the transcription factor, indispensable for the therapeutic exploitation of the ABCs and their relative populations.

We have already mentioned that B cell-intrinsic T-bet depletion had a beneficial effect in murine models of SLE.^34^ Also, we have briefly described a molecular pathway, involving SWEF proteins, that regulates ABC formation,^32^ and we introduced the possibility of targeting and/or enhancing key molecules of the pathway, in an effort to delay or even cancel the generation of ABCs and eventually assuage the symptoms and the activity of SLE. Similarly, since IFNγ and IL-21 promote the differentiation of ABC-like CD11c^hi^T-bet+ B cells,^28,31^ targeting the IL-12-STAT4 axis, the most potent inducer of IFNγ and IL-21 secretion by human CD4+T cells,^49^ may also prove a successful therapeutic approach.

The current therapies for autoimmune diseases are based on immunosuppressive drugs,^50^ which globally affect the immune system and thus increase the risk of infections and cancer.^51^ In order to benefit the patients, targeted approaches need to be introduced to the clinical practice. In the case of T-bet, for example, IL-4 is a well-known antagonist used for silencing its expression.^28^ However, if IL-4 was administered to patients, the potential positive effects on ABCs should be carefully considered and other less, desirable effects, such as the inefficient regulation of the migration of MZ B cells that secrete the anti-inflammatory IL-10 and thus contribute to the remission of collagen-induced arthritis, taken under consideration.^52^

## ABBREVIATIONS

ABCs: Age-associated B cells
ASC: Antibody-secreting cells
BLyS/BAFF: B lymphocyte stimulator/B cell activating factor
CytoFOX: Cytoplasmic FOXO1
DNs: Double negative B cells
DN1: Double negative 1
DN2: Double negative 2
FO: Follicular
GC: Germinal centre
IRF5: Interferon regulatory factor
5 MS: Multiple sclerosis
MZ: Marginal zone
RA: Rheumatoid arthritis
SLC: Surrogate light chain
SLE: Systemic lupus erythematosus
SLEDAI: SLE disease activity index
